# Using Photovoice and Asset Mapping to Inform a Community-Based Diabetes Intervention, Boston, Massachusetts, 2015

**DOI:** 10.5888/pcd13.160160

**Published:** 2016-08-11

**Authors:** Jana Florian, Nicole M. St. Omer Roy, Lisa M. Quintiliani, Ve Truong, Yi Feng, Philippe P. Bloch, Zlatka L. Russinova, Karen E. Lasser

**Affiliations:** Author Affiliations: Nicole M. St. Omer Roy, Yi Feng, Boston Medical Center, Section of General Internal Medicine, Boston University School of Medicine, Boston, Massachusetts; Lisa M. Quintiliani, Ve Truong, Karen E. Lasser, Boston Medical Center, Section of General Internal Medicine, Boston, Massachusetts; Philippe P. Bloch, Zlatka L. Russinova, Boston University, Center for Psychiatric Rehabilitation, Boston, Massachusetts.

## Abstract

**Introduction:**

Diabetes self-management takes place within a complex social and environmental context.  This study’s objective was to examine the perceived and actual presence of community assets that may aid in diabetes control.

**Methods:**

We conducted one 6-hour photovoice session with 11 adults with poorly controlled diabetes in Boston, Massachusetts.  Participants were recruited from census tracts with high numbers of people with poorly controlled diabetes (diabetes “hot spots”).  We coded the discussions and identified relevant themes.  We further explored themes related to the built environment through community asset mapping.  Through walking surveys, we evaluated 5 diabetes hot spots related to physical activity resources, walking environment, and availability of food choices in restaurants and food stores.

**Results:**

Community themes from the photovoice session were access to healthy food, restaurants, and prepared foods; food assistance programs; exercise facilities; and church.  Asset mapping identified 114 community assets including 22 food stores, 22 restaurants, and 5 exercise facilities.  Each diabetes hot spot contained at least 1 food store with 5 to 9 varieties of fruits and vegetables.  Only 1 of the exercise facilities had signage regarding hours or services.  Memberships ranged from free to $9.95 per month.  Overall, these findings were inconsistent with participants’ reports in the photovoice group.

**Conclusion:**

We identified a mismatch between perceptions of community assets and built environment and the objective reality of that environment. Incorporating photovoice and community asset mapping into a community-based diabetes intervention may bring awareness to underused neighborhood resources that can help people control their diabetes.

## Introduction

Diabetes is a serious public health concern. Despite the recent plateau in diabetes prevalence, many people are not achieving clinical goals for diabetes control (1,2). Effective control requires both medical interventions and self-management, including healthy eating, physical activity, and stress reduction, facilitated by self-efficacy and social support (3). Many studies have made associations between risk factors for cardiometabolic disease and the built environment, which includes access to healthy food and physical activity resources (4). Qualitative studies have explored possible mechanisms for these interactions as well as other barriers to effective management (5,6). Current recommendations state that interventions for diabetes management should consider the interplay of individual, family, social, and community factors (7); however, studies tend to be limited to one of these categories.

A better understanding of the perceived value, awareness, and presence of community resources for diabetes management may help inform a community-based diabetes intervention. We combined community asset mapping, the systematic documentation of resources in the environment (8,9), and photovoice, a participatory action research method that engages participants in reflecting on issues in their community through a specific photographic method (10,11). We conducted photovoice and asset mapping sequentially. We used photovoice to generate hypotheses about community factors that affect diabetes self-management and then conducted asset mapping to support or refute these hypotheses. To our knowledge, these methods were not combined previously to provide complementary data on the relationship between subjective perspectives about diabetes self-management and relevant resources in the community.

The aim of this study was to 1) use photovoice to identify factors that contribute to the management of diabetes in census tracts with high numbers of people with poorly controlled diabetes (diabetes “hot spots”) and 2) conduct asset-mapping related to community-level themes that emerged in the photovoice discussions.

## Methods

Photovoice is a community-based participatory research method in which participants use photography to describe their lived experiences related to a certain topic, in this case diabetes management. In our study, we engaged patients from census tracts in the Boston area with 20 or more people with poorly controlled diabetes (ie, hemoglobin A1c levels >9) who received primary care at Boston Medical Center. These census tracts are referred to as diabetes “hot spots.” In previous work, we geocoded registry data of the general internal medicine practice at Boston Medical Center, the largest safety-net hospital in New England. We identified 13 diabetes hot spots (12); the photovoice session elicited the perspectives of a selected group of patients that live in these hot spots.

### Participants and procedure

We obtained institutional review board approval for this research through Boston Medical Center. Eligible participants were at least 18 years old, had diabetes and a hemoglobin A1c level greater than 9, were included in Boston Medical Center’s General Internal Medicine Patient-Centered Medical Home registry, resided in a diabetes hot spot, had telephone access, and spoke English. We sent participants a letter of invitation signed by their primary care provider and then contacted them by telephone to describe the study and answer questions. We contacted 79 patients; 14 declined to participate, 48 could not be reached (eg, did not answer), and 17 agreed to participate. Eleven patients attended the photovoice session.

In April 2015, participants attended a 6-hour photovoice session at a public library in a centrally located diabetes hot spot. A senior research coordinator with extensive experience facilitating photovoice groups led the session. The photovoice prompts and questions used in the session were formulated and agreed upon a priori by the research team, which included a primary care physician (K.L.) and behavioral scientist (L.Q.) with expertise in health disparities and health behaviors and experts in photovoice (Z.R., P.B.).

Before their arrival, participants reviewed the informed consent document with a research assistant over the telephone. Participants signed the document at the beginning of the photovoice session and completed a demographic questionnaire. Next, the participants learned about the photovoice method and relevant ethical and safety guidelines (eg, obtaining consent from people they photographed). The facilitator then led the group in a discussion about photographs of fresh produce, hamburgers and French fries, equipment in a gym, and a row of storefronts, and guided participants in a discussion of the effect of their environment on diabetes self-management. Following the training, each participant received a digital camera and left the library to take pictures that responded to 3 prompts: 1) “From your perspective, what is it like to have diabetes?”, 2) “What gets in the way of your controlling your diabetes?”, and 3) “What could motivate you to control your diabetes?” Participants then returned the cameras to the research team.

Each participant selected the photographs they wanted to share with the group and wrote accompanying narratives for each photograph. These photographs were projected on a large screen for the group to view. The facilitator asked each participant to talk about their photographs, using the following questions to gain insight into the patients’ experience of diabetes: “What is happening in your picture?” “Why did you take a picture of this?” “What does this picture tell us about your life?” “How can this picture provide opportunities for us to improve life?” After each participant presented a picture, the facilitator elicited other members’ perspectives on the photograph.

### Analysis

Discussions were audio-recorded and transcribed. Three research team members (J.F., N.S., Y.F.) independently read each transcript. Team members then developed a set of codes by using levels of the socioecological model as a framework (13). The entire team reviewed these codes. The coding framework was then systematically applied to the transcript and narratives, and any discrepancies in coding were resolved through group consensus.

We performed a literature search for tools to assess community resources discussed by the photovoice group. Because we needed a multicomponent assessment, we modified existing survey tools. Our combined tool evaluated physical activity resources, walking environment, food store availability of “diabetes-friendly” foods, and restaurant availability of healthy food choices. The physical activity resource assessment documented signage, cost, hours of operation, features, and incivilities (eg, graffiti) (14). We derived the walking environment and street assessment from the pedestrian infrastructure, bicycle infrastructure, and aesthetics and character sections of the Built Environment and Active Transportation Neighborhood Assessment (15). For the food store assessment, we followed the methods of a study that used a survey tool of foods recommended for people with diabetes developed by the East Harlem diabetes coalition (16). Last, we used the menu review section of the NEMS-R (Nutrition Environment Measures Study in Restaurants), an assessment tool that evaluates the nutrition environment in restaurants (17). Two research assistants pilot-tested the final combined tool in a non–hot spot census tract and further refined the tool before implementation.

In May 2015, we selected 5 diabetes hot spots that contained the highest number of people with poorly controlled diabetes for asset mapping. We performed Internet searches to generate a preliminary list of resources in each hot spot census tract, which included places of worship, fitness clubs and gyms, parks, schools, community centers, community health centers, libraries, community gardens, farmers markets, food pantries, grocery stores, convenience stores, pharmacies, gas stations, restaurants, and Hubway stations (Boston’s public bicycle sharing program). Next, we used ArcGIS software (ESRI) to map community resources in each hot spot census tract. Two research assistants conducted walking surveys to confirm the presence of resources and identify new resources not found in online databases. Each research assistant independently evaluated community resources in each census tract with the combined assessment tool.

## Results

### Photovoice participant characteristics

The photovoice group participants (n = 11) were mostly female (n = 8), had a mean age of 58 years, and represented 5 diabetes hot spots. All participants identified as black or African American. Seven had graduated from high school, and 9 were unemployed. Most participants (n = 10) described themselves as being in fair or poor health. Six participants had a household income of $20,000 per year or less, and 5 were concerned that they would run out of food at home before they had money to buy more.

Themes at the individual, interpersonal, and community levels emerged from the analysis of the 14 photovoice narratives and the group discussion transcript. Individual-level and interpersonal-level themes are not presented because of our focus on the built environment through community asset mapping. Our results are consistent with previous research on individual-level and interpersonal-level factors (18,19).

### Community asset mapping and photovoice perspectives

**Access to healthy food.** Asset mapping identified 114 community assets in 5 hot spot census tracts (Table 1). Photovoice participants noted a lack of healthy foods in their community stores, whereas asset mapping showed that healthy options (eg, fruits, vegetables, low-fat milk) were available. Participants described how factors in their neighborhoods made it difficult to purchase, prepare, and eat healthy foods. When presented with a photo of fresh produce, several participants described the lack of healthy food available at food stores in their community: “When we go to the corner store, all we see is junk food, sweets, sodas, and nothing healthy. And they never carry anything healthy for you. You would have to go to Stop N Shop or a grocery store.” Even when healthy foods were available, participants described the high costs of produce and other healthy foods.

**Table 1:** Mapped Community Assets (n = 114) in 5 Diabetes Hot Spots^a^, Boston, Massachusetts, 2015

Asset	n (%)
Places of worship	25 (21.9)
Fitness clubs and gyms	2 (1.8)
Parks and community gardens	17 (14.9)
Schools	5 (4.4)
Libraries	2 (1.8)
Food stores	22 (19.3)
Restaurants	22 (19.3)
Food pantries	9 (7.9)
Farmers markets	2 (1.8)
Community centers	3 (2.6)
Community health centers	1 (0.9)
Gas stations	2 (1.8)
Hubway bike stations^b^	2 (1.8)

a Census tracts with 20 or more people with poorly controlled diabetes (hemoglobin A1c >9).

b Boston’s bicycle sharing program.

Asset mapping demonstrated that most food stores (n = 15) were convenience stores, and only 3 were grocery stores (Table 2). At least one food store in each of the 5 diabetes hot spots carried 5 or more varieties of fruits and vegetables. Nearly every convenience store (n = 15) carried diet soda, and most carried low-fat or nonfat milk (n = 11), fresh fruit (n = 13), fresh vegetables (n = 13), and frozen vegetables (n = 12). However, only 3 stores carried whole wheat bread.

**Table 2:** Characteristics of Mapped Food Stores^a^ (n = 22) in 5 Diabetes Hot Spots^b^, Boston, Massachusetts, 2015

Food Store Characteristic	n
**Store type**	
Grocery store	3
Convenience store	15
Gas station	2
Pharmacy	2
**Products available**	
Cigarettes	17
Low-fat or nonfat milk	17
Diet soda	21
Whole wheat bread (fiber ≥2g per slice)	6
Fresh fruit	17
Fresh vegetables	17
Frozen vegetables	16
Canned tuna	16
**Fruit availability, no. of varieties**	
0	5
1–4	7
5–9	6
≥10	4
**Vegetable availability, no. of varieties**	
0	5
1–4	5
5–9	7
≥10	5

a Establishments that sell food for home preparation and/or consumption.

b Census tracts with 20 or more people with poorly controlled diabetes (hemoglobin A1c >9).

**Restaurants and prepared foods**. Photovoice participants described an unhealthy food environment in community restaurants; this description was consistent with the findings of the asset mapping restaurant assessment. Most participants described the abundant unhealthy food options provided by restaurants. One participant said, “I see a lot of pizza and stores outside. It’s hard because I can’t eat that food.” Asset mapping identified 22 restaurants (Table 3). Most of these restaurants (n = 15) were fast-food restaurants. Some restaurants (n = 7) had salad entrees with lowfat or nonfat dressing, but none had brown rice or whole wheat bread, although 19 restaurants served white rice or bread.

**Table 3:** Characteristics of Mapped Restaurants (n = 22) in 5 Diabetes Hot Spots^a^, Boston, Massachusetts, 2015

Restaurant Characteristic	n
**Restaurant type**	
Sit-down^b^	3
Fast casual^c^	4
Fast food^d^	15
**Products available**	
Salad entrée with low-fat dressing	7
Nonfried vegetables	9
Fresh fruit	1
Diet soda	21
White bread or white rice	19
Whole wheat bread or brown rice	0

a Census tracts with 20 or more people with poorly controlled diabetes (hemoglobin A1C >9).

b A restaurant that offers full table service by wait staff who take your order at the table (17).

c A restaurant that does not offer table service but promises higher quality of food and atmosphere than a fast food restaurant. Patrons may order or pay at a counter, and food is often brought to the table (17).

d A restaurant that sells highly processed food prepared in an industrial fashion with standard ingredients and methodical cooking and production methods; food is often finger food that can be eaten quickly and without cutlery (17).

**Figure 1:**
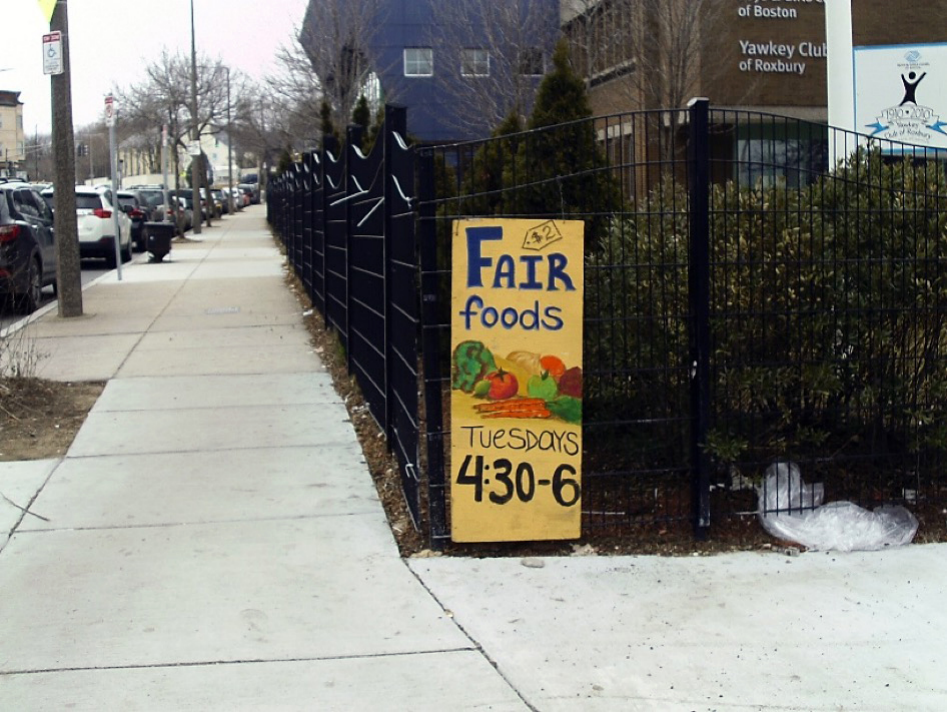
Example of a positive photovoice narrative on food assistance programs. “The picture of the sign lets me know there are healthy food options in my neighborhood that are inexpensive, which is encouraging and promising.”

**Food assistance programs.** Participants provided varying opinions of community food assistance programs. Some described programs positively in their photovoice narratives (Figure 1), but one participant described a program where clients were made to feel ashamed and discouraged from accessing similar programs in the future. Participants also had mixed thoughts on the Supplemental Nutrition Assistance Program (SNAP). Some felt they were able to get enough food with SNAP assistance, and others felt that the amount of assistance was inadequate. Community asset mapping found 9 food pantries. Four of the 5 mapped census tracts had at least 1 food pantry, and 2 of the census tracts had 3 food pantries each. All food pantries were found through online searches, and only 1 food pantry had public signs giving hours of operation.

**Exercise facilities and street assessment**. Photovoice participants described the high cost of accessing indoor exercise facilities, whereas asset mapping showed gymnasiums (gyms) and community centers that had affordable membership options. One participant described how she was laid off from a job that provided a discounted gym membership, and she could no longer afford a membership. Most participants stressed that they knew exercise was important but that cost, family obligations, and joint pain made it difficult to exercise. Three of the census tracts had at least 1 indoor exercise facility (gym or community center). Only 1 of the 5 facilities had signage that indicated the hours or services. Memberships ranged from free to $9.95 per month. Our survey of the pedestrian environment showed that sidewalks were present on both sides of the street and were in excellent or good condition in all census tracts. However, none of the census tracts had adequate street furniture (eg, benches, trash cans). Only 1 census tract had bicycle lanes on all major streets, and none of the census tracts had bicycle racks on major streets.

**Figure 2:**
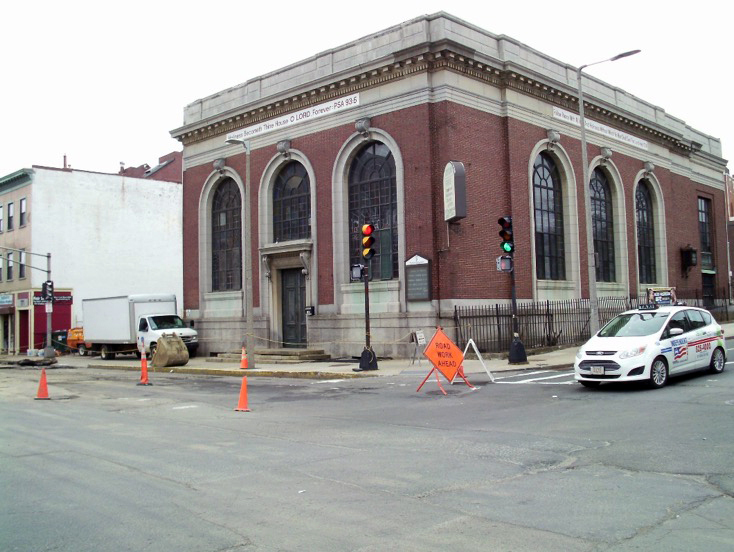
Example of a photovoice narrative on religion, spirituality, and churches: “While viewing the diabetes . . . as a sick component of the body, the church reminds me that I must do what is best to manage this sickness, as the church does what it must to help manage my spiritual needs and well-being.”

**Religion, spirituality, and churches**. Photovoice participants described the important role of church in their lives; asset mapping showed that places of worship were the most abundant community resource in diabetes hot spots. Participants most often discussed religion and church as a method to mitigate stress: “My church is really what drives everything home for me. It’s my safe haven. Even with all the different types of anxiety and stresses that may come in my life, I try to keep [them] at bay.” Several participants described a desire to return to church after hearing other group member’s comments. In a photovoice narrative (Figure 2), one participant described how religion helped her manage her diabetes. Community asset mapping indicated an abundance of churches; 22% of mapped assets were places of worship. Every census tract had at least 3 churches, and one census tract had 10.

## Discussion

The combined use of photovoice and community asset mapping is a novel approach to understanding social and environmental factors that influence diabetes self-management. Photovoice provided information on how participants perceived their environment, whereas community asset mapping quantified environmental characteristics that could be leveraged to facilitate diabetes management. Comparing these data allowed us to identify where perceptions and objective findings converged and diverged. Overall, we found that perceptions and objective measures of churches and restaurants converged, and perceptions and objective measures of food stores and exercise facilities diverged.

A growing body of literature examined the effects of the local food environment on food purchasing, fruit and vegetable intake, and obesity (20,21). Much of this work has focused on food deserts — urban areas without access to healthy foods (22). Prior studies demonstrated that the presence of supermarkets in a neighborhood decreased obesity rates and increased food and vegetable consumption, whereas the presence of convenience stores increased obesity rates (21,23). Food desert research has implications for urban planning and policy and calls for placing more supermarkets in densely populated, poor urban areas (24). However, recent work demonstrated that the mere presence of a supermarket does not increase fruit and vegetable intake or lead to decreased obesity rates (25,26).

Food access cannot be measured solely through the objective presence of healthy food or supermarkets in communities, and food access does not translate to positive dietary outcomes and reduced risk of metabolic disease (20). Our results are consistent with those of studies demonstrating a mismatch between perceptions of the local food environment and the objective reality of that environment. Perceptions may incorporate aspects of food access (eg, acceptability, quality, affordability, daily travel patterns) that are not accounted for through objective measures (27).

How the perceived environment, the objectively measured built environment, and physical activity relate to each other is also complicated. The perception of crime has a negative relationship on levels of physical activity regardless of objective crime rates (28), and perceived neighborhood walkability correlated more with participants’ walking habits than objectively measured walkability (29). However, the relationship between perceived environment and physical activity is generally inconsistent, and both objective and perceived measures are associated with levels of physical activity to various degrees (30). Our study did not examine perceived walkability, but participants noted difficulty in accessing affordable exercise facilities. Community asset mapping documented 5 indoor exercise facilities, some with free membership options. Surprisingly, most of these facilities did not have signage indicating the presence of physical activity resources inside. Inadequate advertising by gym and exercise facilities may contribute to the perceived lack of affordable physical activity resources.

Photovoice combined with asset mapping could form the basis of a community-based diabetes intervention. In this study, photovoice enabled participants to document their experience of diabetes and to confront barriers to diabetes control in their personal environment and community. Many participants demonstrated “change talk” (statements about desire or commitment to make a change in behavior) in response to other group members’ photographs. For example, on hearing one participant speak about the stability that her spirituality brings her in times of stress, several other participants stated their intention to reconnect with their faith and attend church. Along with facilitating support from peers, the photovoice method may reinforce internal motivation to make meaningful changes to better manage one’s chronic disease. In addition, incorporating the results of community asset mapping into photovoice discussions may bring awareness to resources in the community that could be helpful in managing diabetes. Assignments that guide participants to identify and map relevant community resources may challenge potential misperceptions about the physical activity and food environment. 

This study has several limitations. We had a small number of participants and conducted only one 6-hour-long photovoice session. Different themes may have emerged if we had conducted multiple groups with a larger number of participants or if the group was able to spend several sessions together. In addition, photovoice participants did not equally represent all of the 13 diabetes hot spots in Boston, and only 7 of the 11 participants resided in 1 of the 5 mapped hot spots. We also adapted several published survey tools to create this study’s assessment tool. 

Despite these limitations, this study is the first to combine photovoice and community asset mapping to inform the development of a community-based diabetes intervention. Our findings demonstrate that the juxtaposition of photovoice and asset mapping may provide rich insight into patient perceptions, opportunities for diabetes education, and community assets that can be leveraged to improve population health.

## Appendix Additional Community-Level Questions and Answers From Photovoice Discussion and Photovoice Narratives

This file is available for download as a Microsoft Word document.
