# Supplementary material for: Using Photovoice and Asset Mapping to Inform a Community-Based Diabetes Intervention, Boston, Massachusetts, 2015
Source: Prev Chronic Dis. 2016 Aug 11;13:E107. doi: 10.5888/pcd13.160160 (PMC4993113; doi:10.5888/pcd13.160160)
Supplement: Supplementary file 1 [file 16_0160_Appendix.docx]

**Appendix. Additional Community-Level Questions and Answers From Photovoice Discussion and Photovoice Narratives**

| Access to Healthy Food |
| --- |
| Facilitator (F): Is paying for food a real problem?  Participant (P): When you look at the reality of eating healthier it’s costly to do that. Especially when you have other children, or people in the house, they may not even want what you have, even though they (food) are healthy. So you’re trying to school down the rest of the family, and they want other stuff, and it can be very expensive to eat right. |
| P: I see produce that is essential for my good health. But in my neighborhood, accessibility for these products are not available. Most neighborhood stores don’t carry healthy food choices. We need to have stores in my neighborhood to have these produce available for everyone. |
| Restaurants and Prepared Foods |
| P: I wish I could eat Chinese food but I just gotta stay away from it. I live right next door to a Chinese restaurant and it’s hard just to walk by. |
| P: I took that picture because the foods they served around the counter there, I can no longer eat them. The pastas, pizzas, all the subs and all the different sodas that my body can’t deal with anymore so that’s why I took this picture. |
| Food Assistance Programs |
| F: I heard there was a program…where you can get a voucher for fresh fruits and vegetables.  P: I heard it through someone but I don’t think it’s offered to just any and every one. A lot of these places, they’re usually for people who come all the time…I went to the church…to get some help. They took all those that have been coming in and they know who is what. And when you get in there, they make you feel bad. And they give you what crumbs they got leftover… I didn’t go back after, the way they treated us like that…You need to help the people who really need the food. You wouldn’t come into those lines and you humiliate yourself if you didn’t really need them. |
| P: People get food stamps but it’s not always enough. Some people get more money and other some people get less. |
| Exercise Facilities and Street Assessment |
| P: Exercise is the most important thing to me but unfortunately I am not able to maintain it. I can see myself exercising my way to good health. It is very important to do this for my overall health but it’s a challenge for me because financially I cannot maintain this type of thing on a regular basis. Going to the YMCA would be a wonderful benefit if your health insurance could cover it. |
| P: The best option I had was when I was working and the company had a thing with YMCA where they employees could go there and it would only cost $32 a month and it was easy for me to do that. And we also had a group that would walk around the area. My blood glucoses and everything stayed down very low. But when I got laid off, all of that had to change. |
| Religion, Spirituality, and Churches |
| P: I like the picture too because I’m Christian and I go to church. God means everything to me. I believe that a lot of people don’t go to church but they believe and that’s good too. |
| P: After looking at the picture I’ve made my mind to start going to church. I’ve waited for so long. |
